# Constructing a Multiple Sclerosis Diagnosis Model Based on Microarray

**DOI:** 10.3389/fneur.2021.721788

**Published:** 2022-01-20

**Authors:** Haoran Li, Hongyun Wu, Weiying Li, Jiapei Zhou, Jie Yang, Wei Peng

**Affiliations:** ^1^College of Traditional Chinese Medicine, Shandong University of Traditional Chinese Medicine, Jinan, China; ^2^Department of Neurology, Affiliated Hospital of Shandong University of Traditional Chinese Medicine, Jinan, China; ^3^Department of Comprehensive Surgery, Weifang Maternal and Child Health Hospital, Weifang, China

**Keywords:** multiple sclerosis, diagnosis model, microarray, Random Forest, logistic regression, Area Under Curve, Calibration Curve, McDonald Criteria

## Abstract

**Introduction:**

Multiple sclerosis is an immune-mediated demyelinating disorder of the central nervous system. Because of the complexity of etiology, pathology, clinical manifestations, and the diversity of classification, the diagnosis of MS is very difficult. We found that McDonald Criteria is very strict and relies heavily on the evidence for DIS and DIT. Therefore, we hope to find a new method to supplement the evidence and improve the accuracy of MS diagnosis.

**Results:**

We finally selected GSE61240, GSE18781, and GSE185047 based on the GPL570 platform to build a diagnosis model. We initially selected 54 MS susceptibility locus genes identified by IMSGC and WTCCC2 as predictors for the model. After Random Forests and other series of screening, the logistic regression model was established with 4 genes as the final predictors. In external validation, the model showed high accuracy with an AUC of 0.96 and an accuracy of 86.30%. Finally, we established a nomogram and an online prediction tool to better display the diagnosis model.

**Conclusion:**

The diagnosis model based on microarray data in this study has a high degree of discrimination and calibration in the validation set, which is helpful for diagnosis in the absence of evidence for DIS and DIT. Only one SLE case was misdiagnosed as MS, indicating that the model has a high specificity (93.93%), which is useful for differential diagnosis. The significance of the study lies in proving that it is feasible to identify MS by peripheral blood RNA, and the further application of the model and be used as a supplement to McDonald Criteria still need to be trained with larger sample size.

## Introduction

Multiple sclerosis (MS) is an immune-mediated demyelinating disorder of the central nervous system (CNS). The most frequently involved parts of the disease are periventricular white matter, optic nerve, spinal cord, brainstem, and cerebellum, characterized by limb weakness, sensory abnormalities, ataxia and visual, and cognitive changes. The pathological mechanism of MS is still unknown, but its occurrence is closely related to autoimmunity and environmental factors (1). MS usually occurs in young adults, and it is more common in women (2). MS has a great impact on the motor function and economy of early adult life, thus significantly reducing the quality of life. Disease-modifying treatments have mostly failed as treatments for progressive multiple sclerosis (3). However, early diagnosis and treatment are still very effective in reducing recurrence and disability rates in relapsing-remitting MS (RRMS).

The diagnosis of MS is based on symptoms and signs of the central nervous system, as well as CNS demyelination evidence for dissemination in space (DIS) and dissemination in time (DIT). The detection of MRI and cerebrospinal fluid (CSF) has greatly improved the accuracy of diagnosis and differential diagnosis of MS from other diseases, but there were still some misdiagnoses (4). At present, an increasing number of studies have found that the biomarkers in blood have obvious specificity in patients with MS (5, 6). The International Multiple Sclerosis Genetics Consortium (IMSGC) and the Wellcome Trust Case Control Consortium 2 (WTCCC2) identified more than 50 MS susceptibility loci in genome-wide association studies (GWAS) with a large sample size (*n* = 9,772) (7). With the increasing maturity of microarray technology, economical and convenient gene detection makes the gene diagnosis of MS possible. In this study, the probe microarray data of blood samples of MS, healthy control, and other inflammatory CNS diseases were obtained from the Gene Expression Omnibus (GEO) database, and an MS diagnosis model based on gene expression was constructed.

## Results

### Data Processing

**Figure 1:**
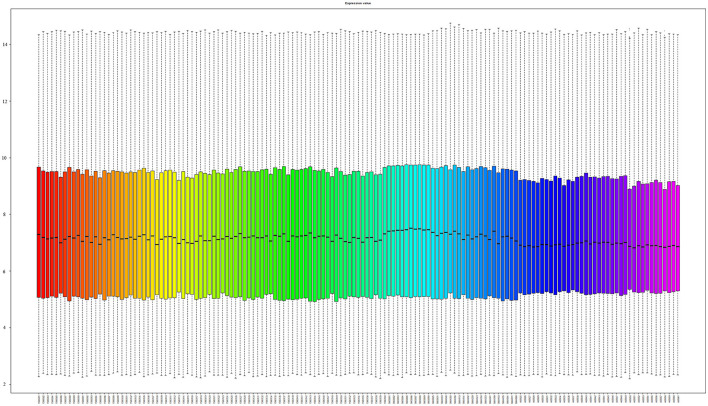
Boxplot of 147 gene chips. The median gene expression of 147 samples is almost at the same level.

We finally selected GSE61240, GSE18781, and GSE185047 based on the GPL570 platform (Affymetrix Human Genome U133 Plus 2.0 Array) to build a diagnosis model. We unified the raw probe data of the three series to get the matrix data of gene expression. We have drawn a boxplot based on gene expression, which shows that 147 arrays are at the same expression level, and there was no systematic error between the three series (as shown in Figure 1 and Supplementary Table 1). One hundred and forty-seven arrays in the above three series were divided into a training set and a validation set. The training set contains samples of 39 RRMS (one RRMS was eliminated after inspection), 6 sarcoidosis, 10 systemic lupus erythematosus (SLE), and 18 healthy control. The validation set contains samples of 40 RRMS, 6 sarcoidosis, 10 SLE, and 17 healthy control. The training set is used to construct the prediction model, and the validation set is used to verify the accuracy and reliability of the model prediction (as shown in Supplementary Tables 2, 3).

### Screening Predictors of the Model

**Figure 2:**
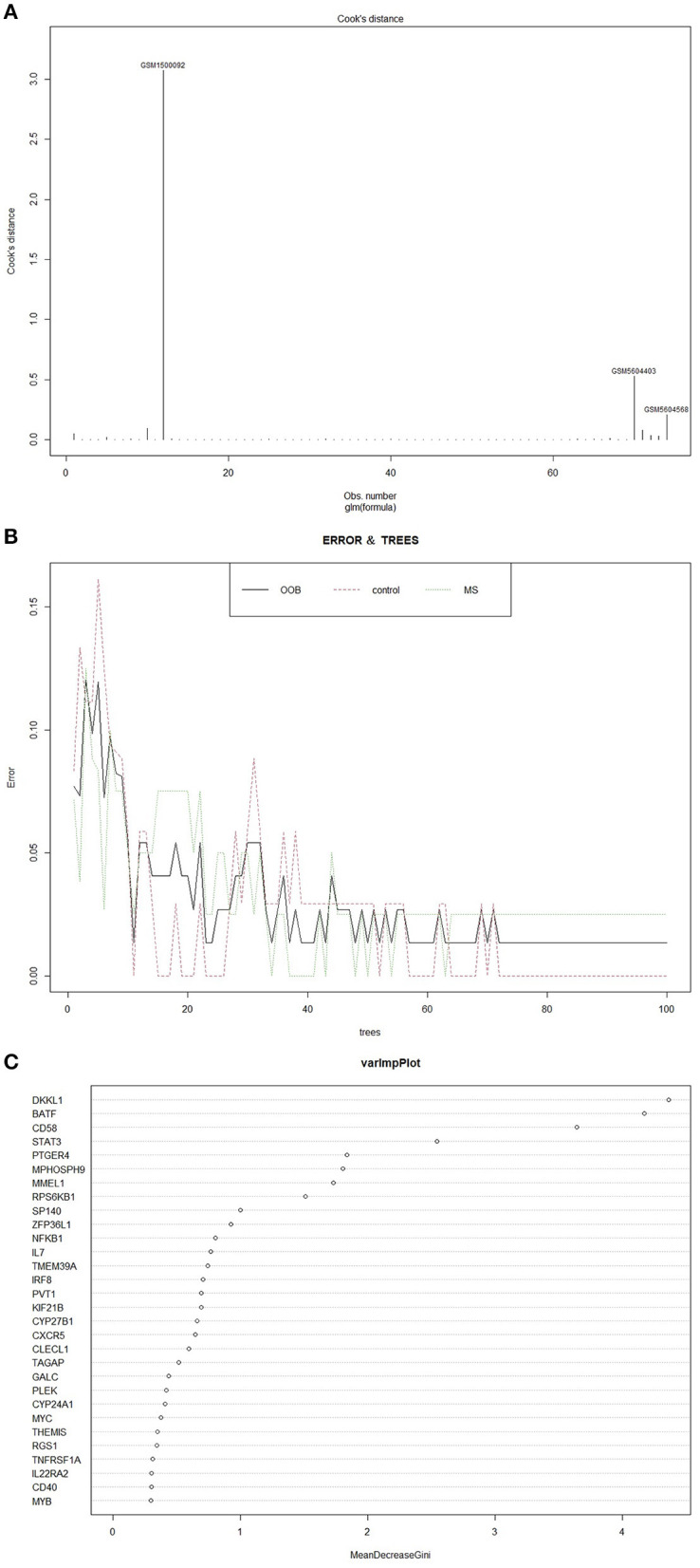
**(A)** Cook's Distance describes the influence of a single sample on the entire regression model. The greater the Cook's distance, the greater the influence. **(B)** The relationship between the number of classification trees and the error in Random Forests. With the increase of the number of classification trees, the classification error gradually decreases, and the model gradually tends to be stable. **(C)** Gini index of predictors in the model, which reflects the importance of predictors.

We adopted the conclusions of the MS genome-wide association studies of IMSGC and WTCCC2 and used 54 MS susceptibility locus genes as the predictors preliminarily included in the model (7). Subsequently, we rigorously screened 54 predictors using Random Forests and obtained 8 predictors with a Gini index greater than 1 (as shown in Figure 2) (8, 9). We used the restricted cubic spline (RCS) to test the non-linearity of the 8 predictors and found that CD58 and MMEL1 did not satisfy the linear relation with the dependent variable while the *P*-value is 0.0041 and 0.0492 (*P* < 0.05), so both predictors were eliminated. Multicollinearity refers to the high correlation between variables in the linear regression model, which makes the model difficult to estimate accurately. We used variance expansion factors (VIF) to evaluate the multicollinearity of variables (10). The VIFs of STAT3 and RPS6KB1 were greater than 5 and were eliminated, and it was considered that there was multicollinearity between them. Finally, we selected 4 genes (DKKL1, BATF, PTGER4, MPHOSPH9) as final predictors of the model and used them in the subsequent model construction.

### Model Establishment and Evaluation

**Figure 3:**
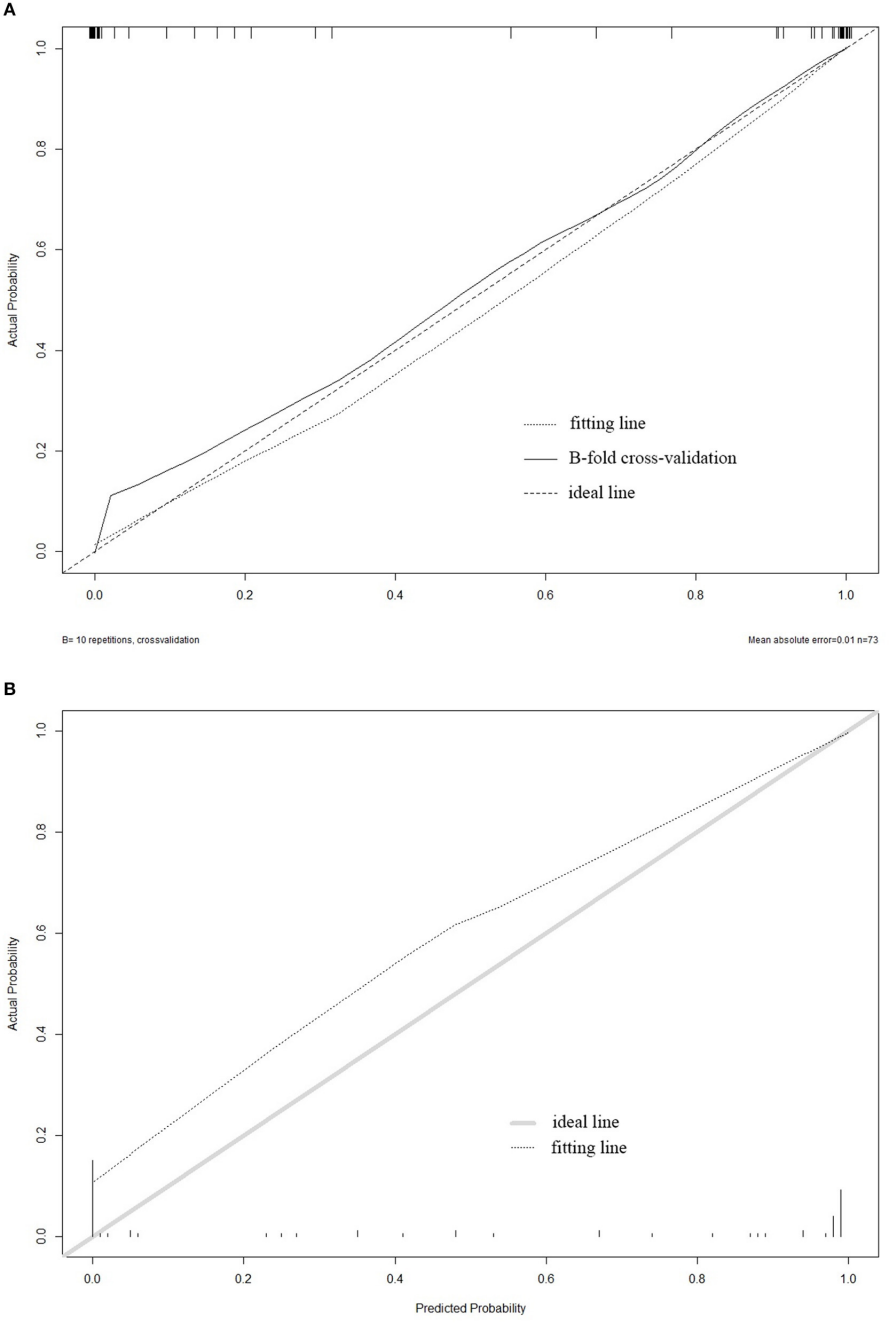
**(A)** The Calibration Curve of the training set. **(B)** The Calibration Curve of the validation set. The abscissa of the graph is the predicted probability, and the ordinate is the actual probability. The ideal line indicates that the actual probability and the predicted probability are perfectly coincident under an ideal situation. The fitting line represents the predicted probability corresponding to the actual probability. If the predicted probability is greater than the actual probability, that is, the risk is overestimated, then the fitting line is under the ideal line. If the predicted probability is less than the actual probability, that is, the risk is underestimated, then the fitting line is above the ideal line. The graph also shows the performance of the model in B-fold cross-validation (*B* = 10).

**Figure 4:**
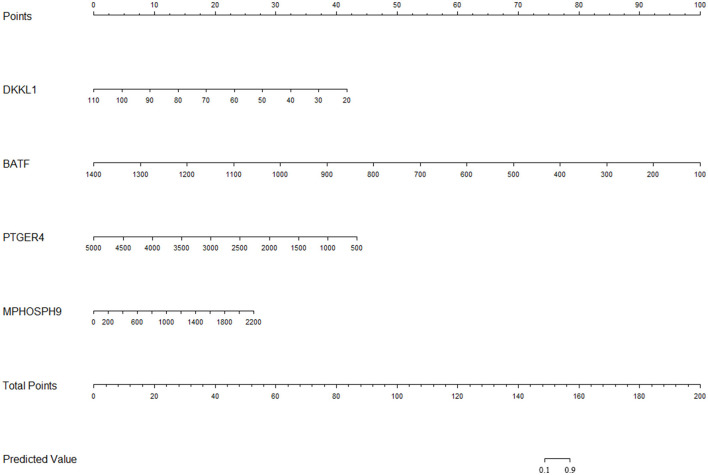
The nomogram of the diagnosis model. Nomogram is a simple tool to calculate the output probability of the model. For more accurate probability calculation, please refer to the online prediction tool.

The influential point in the data has a great influence on the stability and authenticity of parameter estimation. Therefore, this study used Cook's distance to evaluate the influential point in the data. When the Cook's distance is less than 1, it is considered that there is no influential point (11). After inspection, the Cook's distance of sample GSM1500092 is greater than 3, so GSM1500092 was excluded (as shown in Figure 2). The actual clinical manifestations of the diagnosis model constructed according to the training set should refer to its prediction accuracy in the independent validation set. We used the diagnosis model to predict the training set and validation set and evaluated the accuracy of the model according to the discrimination and calibration. The original training set and B-fold cross-validation were predicted, respectively. The C-statistics of the diagnosis model in the original training set and in B-fold cross-validation were both 0.99. The Calibration Curve drawn with R can directly indicate that the diagnosis model has a high calibration in the original training set, and also has a good performance in B-fold cross-validation (as shown in Figure 3). Subsequently, the diagnosis model was used to predict the validation set, and the receiver operating characteristic curve (ROC) and a Calibration Curve were drawn (as shown in Figure 3). The Area Under Curve (AUC) is 0.96, and the performance of the model in the Calibration Curve is close to that in the B-fold cross-validation of the training set. After determining that the diagnosis model has a good performance, we used the final model to build a nomogram (as shown in Figure 4) and an online prediction tool (https://acireman.shinyapps.io/dynnomapp/).

**Figure 5:**
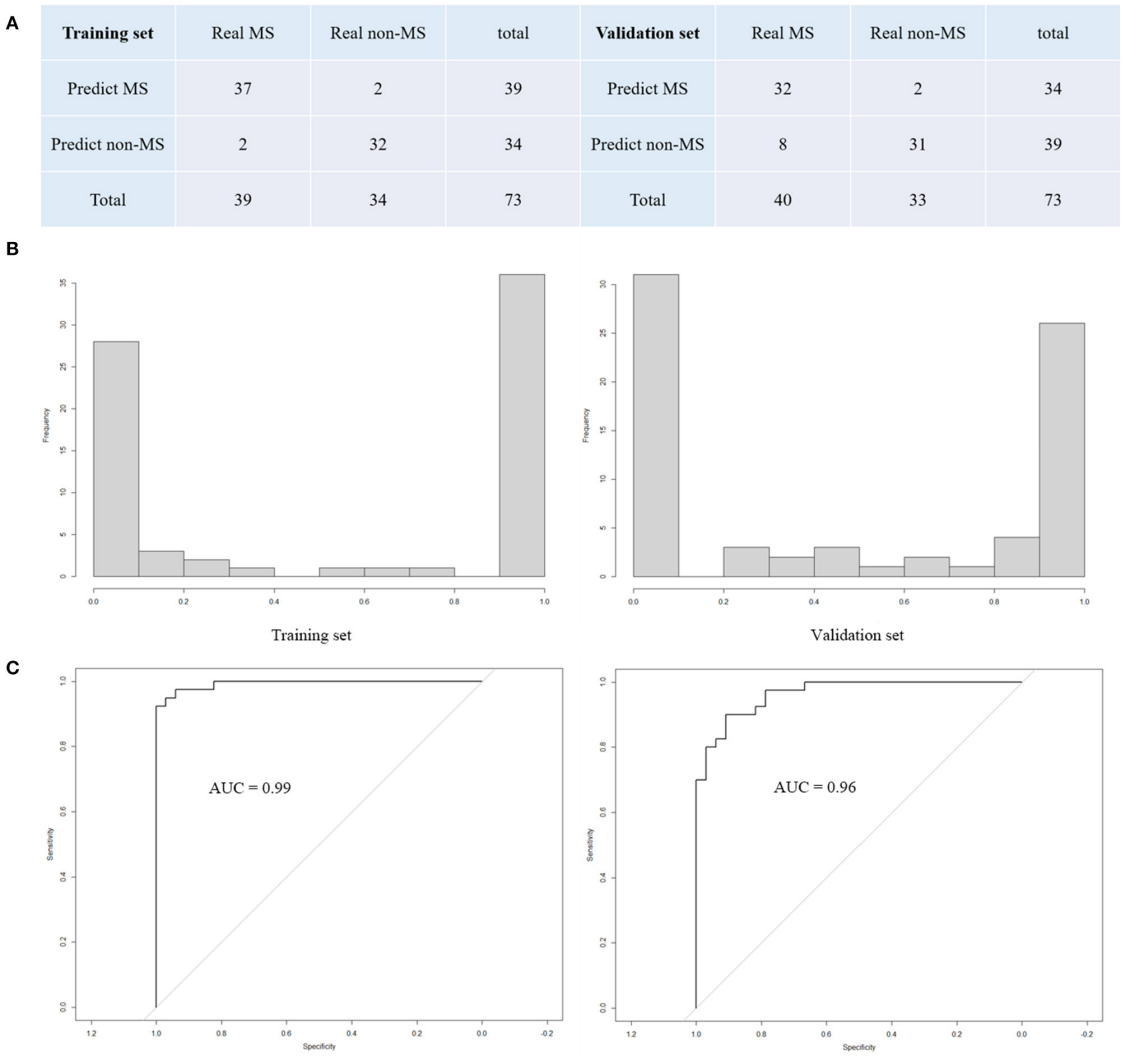
**(A)** The confusion matrix of the prediction model. **(B)** The histogram of the output predicted probability, and it can be seen that the distribution of the predicted probability is concentrated at both ends. **(C)** The receiver operating characteristic curve (ROC). AUC is the size of the area under ROC.

We have drawn a histogram of the output probability, and it can be seen that the distribution of the predicted probability is concentrated at both ends (as shown in Figure 5). In order to show the prediction accuracy of the model, we drew a confusion matrix based on all samples in the validation set (as shown in Figure 5). With the probability threshold set to 0.5, 63 out of the 73 samples were correctly classified, with an accuracy of 86.30%. Eight cases of RRMS were wrongly judged as non-MS, one case of healthy control and one case of SLE were wrongly judged as RRMS with a sensitivity of 80.0%, specificity of 93.93%, positive prediction value (PPV) of 94.11%, negative prediction value (NPV) of 79.48%. Similarly, we also drew a confusion matrix for the prediction of the model in the training set (as shown in Figure 5).

## Discussion

The McDonald Criteria in 2017 is very strict and complicated. Firstly, it is suitable for patients with a typical clinically isolated syndrome (CIS) (12). Secondly, evidence of DIS and DIT are required to confirm the diagnosis, which refers to the multiple locations of lesions and the relapsing-remitting course (13, 14). Therefore, the difficulties of McDonald Criteria lie in three aspects: identifying demyelinating lesions of the CNS, identifying different positions of the lesions, and identifying the course of relapse and remission (or coexistence of old and new lesions).

The current development of imaging greatly improves the diagnosis of MS. For example, susceptibility-weighted sequences at 3T can identify the paramagnetic rim lesions and central vein sign (15, 16), which are the characteristic lesions of MS. However, since the related devices have not been widely used in clinical practice and the difficulties to determine the threshold for their diagnosis (17), they are only recommended as differential diagnostic markers, but not for clinical apply (18). Aside from neuromyelitis optica spectrum disorders (NMOSDs), non-specific MRI findings of common diseases (such as age-related vascular white matter lesion and migraine with a single periventricular lesion, which is not uncommon) are the most common misdiagnosis of MS (12). Therefore, the specificity of MRI in differentiating demyelinating lesions cannot meet clinical needs.

It is sometimes difficult to find evidence of lesions for DIS and DIT. Although both cortical lesions and juxtacortical lesions can be used as evidence of DIS, the ability of MRI to identify demyelinating lesions of cortex and distinguish them from other cortical lesions is limited at present. As evidence of DIS, the number of paraventricular lesions has been changing for years. Although the 2016 MAGNIMS Criteria found that the specificity of a single paraventricular lesion was low (19), the 2017 McDonald Criteria still used a single paraventricular lesion as evidence of DIS, which improved the sensitivity but reduced the specificity (12). Because of the lack of clinical symptoms or MRI (spinal cord MRI is not usually listed as routine imaging), the lesions in the spinal cord may be neglected. In the absence of contemporaneous or current objective evidence, historical events should be carefully accepted. Especially for patients with a first demyelinating attack or primary-progressive MS, the lack of evidence of DIT will affect the definitive diagnosis and the beginning of long-term disease-modifying treatment.

Based on the above objective restrictions, McDonald Criteria in 2017 suggests that CSF-specific oligoclonal bands (OCB) can be used as evidence of DIT, and suggests that spinal cord MRI should be performed as soon as possible in the absence of evidence or typical clinical manifestation (12). Similarly, we hope to develop a new dimension of evidence as a supplement when DIS or DIT evidence is insufficient under McDonald Criteria, even as a differential diagnosis of MS from other inflammatory demyelinating diseases or other CNS diseases. The MS genome-wide association studies of IMSGC and WTCCC2 identified more than 50 susceptibility locus genes among 9,722 cases of European descent (7), which makes it possible to identify MS from a genome.

On the other hand, brain MRI is not accurate for evaluating subcortical demyelination and spinal cord MRI is not as sensitive as the brain in detecting lesions (20), while CSF OCB has received more and more attention in the diagnosis of MS, and the lack of OCB has a very high negative predictive value, indicating other diagnoses should be considered (21). The detection of aquaporin-4 (AQP4) antibody can be used to distinguish MS and NMOSDs (22). For MS with the complexity of etiology, pathology, clinical manifestations, and the diversity of classification, it may be difficult to diagnose from a single aspect, and the combination of multiple evidence will improve the accuracy of the diagnosis. The inspiration for us is, a single gene may not be able to diagnose MS, but the diagnostic model based on the expression of multiple genes can significantly improve the accuracy.

At present, the auxiliary examination of MS mainly involves MRI, CSF electrophysiological evidence, and CSF immunological evidence. The blood biomarkers and the evidence based on peripheral blood gene expression have not reached a consensus in the diagnosis of MS. However, as an invasive examination, lumbar puncture increases the risk of hemorrhage and infection and has many complications such as cerebral hernia and low intracranial pressure. In addition, the blood sample is more inexpensive, more convenient to obtain, and more acceptable than a CSF sample. Therefore, based on the peripheral blood gene expression, the diagnosis model of MS was established, which provides a new dimension and a supplement for the diagnosis of MS.

At present, there are many popular methods applied to the diagnosis model, including support vector machine, artificial neural network, logical regression, and so on. Compared with the other model (23), the reason for choosing logistic regression in this study is that the linear regression model can output linear predicted probability, so as to provide a reference for clinical diagnosis. And the prediction accuracy is similar to other models. In addition, the nomogram and online prediction tool based on the logistic regression model can provide convenience for clinical diagnosis and are closely combined with the selected gene expression. The predicted probability of MS can be output by inputting the expression of 4 genes into the online prediction tool. Both machine learning and the logistic regression model selected in this study needed large data to train a model with excellent classification and prediction function. At present, the sample size of a single microarray in GEO is often small. Therefore, most bioinformatics research adopted the method of combining microarray data from different data sets (but based on the same platform) to increase the sample size. For example, the SVA package of R is often used to remove the batch effect of the microarray from different data sets, which is widely used in data mining (24). However, eliminating the batch effect will inevitably modify the probe expression in the raw data as a whole. Whether there is a significant difference between the final model with the modified data as the training set and the final model with the raw data, and which of the final prediction accuracy of the two methods is better, the relevant literature support has not been found. Therefore, we chose to analyze the raw data of 147 gene chip arrays from three series in a unified way, so as to reduce the systematic error caused by directly merging the microarray matrix and the correction of batch effect.

BATF is a transcription factor that regulates IL-17 expression and Th17 differentiation. And early growth response gene-2 (Egr-2) is an intrinsic regulator that controls Th17 differentiation by inhibiting BATF activation, which may be important in controlling the development of multiple sclerosis (25). Dickkopf (Dkk) gene includes four members of a small gene family (Dkk1-4) and a unique Dkk3-related gene DKKL1, which plays an important role in vertebrate development, and they locally inhibit Wnt regulated process. In adults, Dkks is related to bone formation and bone diseases, various tumors (including gliomas), and Alzheimer's disease (26). Although the expression of DKKL1 is necessary for normal nerve development, the over-expression of DKKL1 is the characteristic of many neurodegenerative diseases, such as stroke, Alzheimer's disease, Parkinson's disease, and temporal lobe epilepsy (27), and it can play an important role in pathological conditions such as tumorigenesis and cancer progression (28). Prostaglandin E2 receptor EP4 subtype (PTGER4) is highly correlated with immune response and inflammatory response (29), but its role in MS has not been systematically studied.

Unfortunately, the GPL570 chip does not annotate the probes of HLA-DRB1. However, studies on the relationship between HLA and diseases have shown that the incidence of some diseases is related to the detection rate of some special types of HLA. Most of these patients are diseases with unknown pathogenesis, abnormal immune function, and genetic tendency. Therefore, analyzing the expression of HLA antigen is not only helpful for understanding the pathogenesis but also is of great significance to the diagnosis, prevention, and prognosis of diseases. HLA class II encoded molecules are cell surface glycoproteins whose primary role in an immune response is to display and present short antigenic peptide fragments to peptide/MHC-specific T cells (30). In populations of European descent, allele DRB1^*^15:01 has the strongest association with multiple sclerosis among all HLA class II alleles (7).

The significance of the study lies in proving that it is feasible to identify MS by blood RNA, and the specificity of the model is still relatively high. In the future, it may be possible to combine image evidence, laboratory evidence, and genetic evidence into a diagnosis model based on machine learning. However, the sample size of the diagnostic model is too small for independent diagnosis and other CNS demyelinating diseases were not included in the training set (since no demyelination microarray data other than MS could be found in the GEO database). For further clinical application, it is still necessary to establish a larger sample cohort to include more other CNS diseases, especially inflammatory demyelinating diseases. In addition, the diagnosis model established in this study is only suitable for the gene chip based on the GPL570, which is a limitation for clinical use.

## Conclusion

The diagnosis model based on microarray data in this study has a high accuracy of 86.30% in the validation set, which is helpful for diagnosis in the absence of evidence for DIS and DIT. Only one SLE case was misdiagnosed as MS, indicating that the model has high specificity (93.93%), which is useful for differential diagnosis. The significance of the study lies in proving that it is feasible to identify MS by peripheral blood RNA, and the further application of the model and be used as a supplement to McDonald Criteria still need to be trained with larger sample size.

## Methods

### Data Retrieval Strategy

GEO is a public functional genomics database, which accepts microarray and sequence-based data. We searched with “demyelinating” and “blood” as the keywords, and preferred the data with large sample size, including MS, healthy control, and other inflammatory CNS diseases. In order to ensure the reliable prediction effect of the diagnosis model, we selected a training set to establish the model, and a validation set to verify the discrimination and calibration of the model. Considering the differences in the manufacturing process of chip manufacturers, we must ensure that the series of data comes from the same platform (Affymetrix Human Genome U133 Plus 2.0 Array, which is named GPL570 in GEO).

### Processing of Raw Data

We downloaded the original probe files of GSE 61240, GSE 18781, and GSE 185047, with a total of 147 arrays. We used the RMA function of the R package “affy” to uniformly process the raw data, including background correction, standardization, summarization, and log-transformed. Through the above steps, we have got a matrix based on the expression of probes. Then, we annotated the probe matrix with R package “hgu133plus2.db,” thus transforming the matrix from probe level to gene level. Since the data were log-transformed by the RMA function, we restored the data to facilitate the construction of the linear regression model. Finally, we obtained a microarray matrix with 20,862 rows and 147 columns. It is worth mentioning that there are 3 processed microarray matrixes in the GEO database for the three series. However, after being processed by different senders and batches, the microarray matrixes have a large systematic error, which is not conducive to the model construction and validation. Therefore, we must uniformly process the original 147 gene chip arrays so that the expression levels of the 147 chips of the three series are at the same level (as shown in Figure 1).

### Screening Predictors by Random Forests

According to stratified random sampling, we divided the microarray matrix into a training set and a validation set. The training set and the validation set do not contain repeated samples. We initially selected 55 MS susceptibility locus genes identified by IMSGC and WTCCC2 as predictors for the model. Therefore, we excluded the other genes in the microarray matrix to reduce the amount of computation. Since GPL 570 does not have a probe to annotate TNFRSF6B, there are only 54 genes in the training set and the validation set. After importing data into R, the “status” was transformed into a classification variable and no missing values were found in the data. Subsequently, we further screened the predictors through Random Forests based on the R package “randomForest.” When the classification tree reaches about 100 trees, the classification of Random Forests tends to be stable (as shown in Figure 2). At this point, we got the Gini index of the predictors and screened out the first 8 genes with a Gini index greater than 1.

### Further Screening of Predictors and Establishment of Logistic Regression Model

The restricted cubic spline (RCS) was used for further non-linear tests of the selected predictors. After eliminating the predictors that do not satisfy the monotonicity, only the linear predictors were retained for the establishment of the logistic regression model. We found that there were influential points and multicollinearity in the model, and we excluded the samples with a Cook's distance greater than 1 to ensure that the data in the training set were reasonable. The predictors with multicollinearity were excluded to improve the stability of the model. After multiple screenings, only 4 out of the 55 initial predictors were used to construct the logistic regression model and the logistic regression model was established based on R packages “rms” and “glmnet.”

### Internal and External Validation of the Diagnosis Model

The logistic regression model is a powerful tool for the prediction of clinical events and allows both classified variables and continuous variables to be included in the model. In order to avoid underfitting or overfitting of the model, we carried out internal and external validation of the diagnosis model and measured the accuracy of the prediction results of the model through discrimination and calibration (31). In the internal validation, we used the original training set for validation and then carried out B-fold cross-validation. Before external validation, the data of the validation set was imported into R to identify the missing values and outliers of the data, and the classification variables were transformed into factor form. Finally, the validation set data was used for external validation of the model.

### Establishment of Nomogram and Online Prediction Tool

After confirming that the diagnosis model has excellent prediction performance in both training set and validation set, the nomogram based on the logistic regression model is established by using the R package “rms.” Nomogram is an imprecise calculation tool based on an image. After synthesizing the scores of all predictors, the total score is corresponding to the probability of the prediction result. The nomogram can roughly estimate the probability of disease occurrence of each clinical sample, which is simple and convenient. In order to further increase the accuracy of the prediction results, an online prediction tool was developed based on a dynamic nomogram to facilitate the further verification of the diagnosis model.

## Data Availability Statement

The datasets presented in this study can be found in online repositories. The names of the repositories and accession numbers can be found in the article.

## Author Contributions

WP: conception and design, funding and administrative support. WL and JY: provision of study materials. HL, HW, and JZ: collection and assembly of data. HL: data analysis and interpretation. All authors: manuscript writing and final approval.

## Funding

This work was supported by the Shandong Provincial TCM Cardiovascular and Cerebrovascular Disease Clinical Medicine Research Center (2018103).

## Conflict of Interest

The authors declare that the research was conducted in the absence of any commercial or financial relationships that could be construed as a potential conflict of interest.

## Publisher's Note

All claims expressed in this article are solely those of the authors and do not necessarily represent those of their affiliated organizations, or those of the publisher, the editors and the reviewers. Any product that may be evaluated in this article, or claim that may be made by its manufacturer, is not guaranteed or endorsed by the publisher.
